# Bitstream-Based Neural Network for Scalable, Efficient, and Accurate Deep Learning Hardware

**DOI:** 10.3389/fnins.2020.543472

**Published:** 2020-12-23

**Authors:** Hyeonuk Sim, Jongeun Lee

**Affiliations:** ^1^School of Electrical and Computer Engineering, Ulsan National Institute of Science and Technology, Ulsan, South Korea; ^2^Neural Processing Research Center, Seoul National University, Seoul, South Korea

**Keywords:** bitstream-based neural network, neuromorphic computing, stochastic computing, deep learning hardware, dynamic precision scaling, SC-CNN, variable precision

## Abstract

While convolutional neural networks (CNNs) continue to renew state-of-the-art performance across many fields of machine learning, their hardware implementations tend to be very costly and inflexible. Neuromorphic hardware, on the other hand, targets higher efficiency but their inference accuracy lags far behind that of CNNs. To bridge the gap between deep learning and neuromorphic computing, we present bitstream-based neural network, which is both efficient and accurate as well as being flexible in terms of arithmetic precision and hardware size. Our bitstream-based neural network (called *SC-CNN*) is built on top of CNN but inspired by stochastic computing (SC), which uses bitstreams to represent numbers. Being based on CNN, our SC-CNN can be trained with backpropagation, ensuring very high inference accuracy. At the same time our SC-CNN is deterministic, hence repeatable, and is highly accurate and scalable even to large networks. Our experimental results demonstrate that our SC-CNN is highly accurate up to ImageNet-targeting CNNs, and improves efficiency over conventional digital designs ranging through 50–100% in operations-per-area depending on the CNN and the application scenario, while losing <1% in recognition accuracy. In addition, our SC-CNN implementations can be much more fault-tolerant than conventional digital implementations.

## 1. Introduction

In a broad sense of the term, *neuromorphic system* refers to a system engineered based on the organizing principles of the nervous system (Mead, 1990). For instance, a CMOS transistor's I-V curve follows an exponential curve under specific conditions and the amount of charge in a capacitor is the time integration of current. Thus, if a system's computation mostly consists of the elementary operations directly derived from the physical principles of devices, such as exponential and time integration, extremely efficient systems can be built by using those elementary operations of devices, as opposed to using AND and OR primitives, which is an artifact of digital design principle (Mead, 1990). Along the same line, neuromorphic system also means mimicking the *structure*, in addition to the behavior, of the nervous system, which is argued to be an important ingredient to attaining desirable system properties, such as high energy efficiency and error resilience, which may be as essential as accuracy in biological nervous systems (Hawkins and George, 2006).

On the other hand, neural networks in the neuromorphic camp, exemplified by the spiking neural networks (SNNs) (Lee et al., 2016), are criticized for their low performance; their inference accuracy lags far behind that of artificial neural networks used in deep learning (Roy et al., 2019). This criticism is a serious one, since the deep learning's efficiency, which is generally regarded as low compared with that of SNNs and other neuromorphic-based ones, can be improved a lot, if inference accuracy can be sacrificed. For instance, low precision networks (Zhou et al., 2016) and binary/ternary networks (Courbariaux and Bengio, 2016; Hubara et al., 2017) have far less computation compared with the original models at the cost of slight accuracy loss. One may argue that the apparent performance advantage of deep learning comes partly from the dataset itself, given the pivotal role played by datasets in the evolution of deep learning (Roy et al., 2019). Nevertheless, it is clear that there is a widening gap between neuromorphic computing and deep learning in terms of accuracy, which must be remedied to make the neuromorphic approach more appealing.

To bridge the gap between neuromorphic computing and deep learning, we present a class of neural networks based on bitstreams. Our neuron model is inspired by stochastic computing (SC), which is an alternative design principle for hardware that is distinguished from both digital and analog design principles. In SC, a number is represented as a bitstream that can be carried on a single wire over a period of time, which is reminiscent of analog computing, yet at the same time, SC circuits can be entirely made out of digital components. The advantages of SC over digital implementations, such as low-cost computation (e.g., multiplication), flexible precision, and high error resilience has led to many applications in image processing and neural networks (Kim et al., 2016; Li et al., 2016, 2017a,b; Ren et al., 2016, 2017; Sim et al., 2017; Zhakatayev et al., 2018). However, the conventional SC as applied to neural networks suffers from the low accuracy problem especially with large neural networks, much like neuromorphic computing.

**Figure 1:**
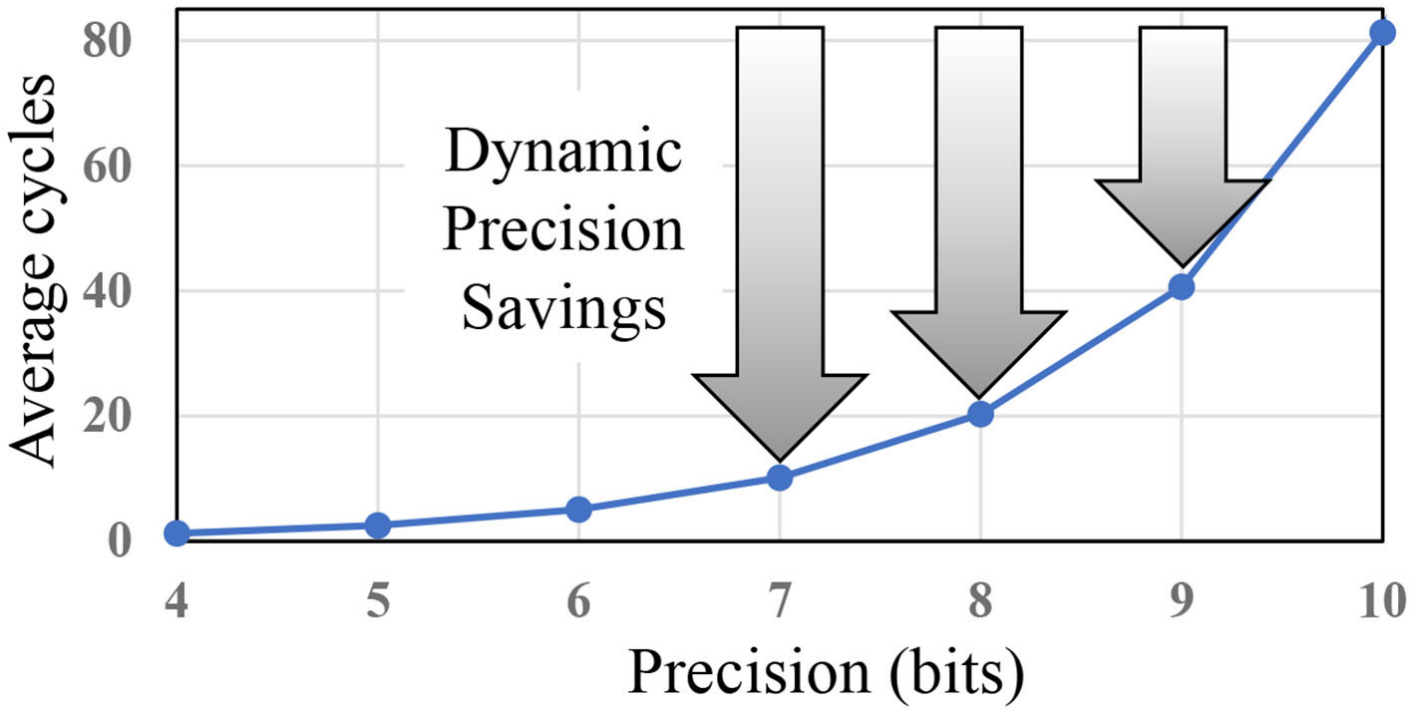
Dynamic precision, or using lower precision whenever possible, can give huge boost in efficiency for stochastic computing (SC).

Our bitstream-based neural network, which is based on convolutional neural network (CNN) and hence called *SC-CNN*, addresses the low accuracy problem of SC while retaining its main advantages. At the same time, our neural network is deterministic, hence repeatable, and is highly accurate and scalable even to large networks^1^. Much like any CNN, our SC-CNN can be trained with backpropagation, ensuring very high inference accuracy. In addition, as CNNs grow more complex and diverse, there is a need for a more reconfigurable hardware architecture that can run various CNNs with different precision requirements at high efficiency. Such reconfigurable precision, which we call *dynamic precision scaling* (DPS), is particularly useful for SC, where 1-bit saving can reduce computation latency by 50% (see Figure 1), suggesting a great potential for higher efficiency on CNNs with diverse precision requirements.

In this paper, we present SC-CNN, which is highly optimized for both accuracy and efficiency as well as flexibility, such as dynamically adjustable precision. Specifically, we first propose dynamic precision scaling for SC-CNN, which extends our previous work (Sim and Lee, 2017) such that the precision of input/output data can be arbitrarily modulated at runtime (see section 3). The extension has very little overhead and allows us to be efficiently parsimonious with regard to precision, which gives a considerable reward in latency saving. Second, in terms of allocating precision across the value range of a variable, we observe that in some layers input activations are always non-negative, meaning that we can reduce precision by 1 bit (with corresponding 50% latency saving) and still get effectively the same accuracy. We call this optimization *half-range specialization* (HRS). Importantly, we implement the HRS optimization as an add-on feature that can be switched on dynamically, so that the same hardware can run all layers of a CNN regardless of the input range (see section 3.4). Third, we present our design methodology to optimize precision across layers of a CNN in section 4. Fourth, exploiting the compactness of our neuron we explore multi-dimensional parallelism to better utilize a given area budget. This is motivated by the fact that the nervous system does no time-multiplexing but implements all neurons with dedicated resources, which is likely an important factor of its high efficiency. We show that multi-dimensional parallelism can give a significant efficiency improvement for SC-CNN over using limited parallelism.

Our experimental results demonstrate that our SC-CNN can be as efficient as conventional digital designs up to ImageNet-targeting CNNs, such as AlexNet (Krizhevsky et al., 2012) and GoogleNet (Szegedy et al., 2015), with <1% degradation in recognition accuracy. This is quite significant as the previous work on SC-CNN was only able to show it up to Cifar-10 (Sim and Lee, 2017), which is much smaller than ImageNet. Second, we show that our SC-CNN can be over 100% more efficient in terms of operations-per-area over conventional digital design when the same hardware is used for multiple CNN applications of varying precision requirements. Third, we show that even for a single application scenario where the hardware is designed only for one application (e.g., AlexNet), our SC-CNN can still be 52% more efficient than conventional digital design. Fourth, we show that our neuromorphic SC-CNN is very scalable, achieving very high efficiency at a high throughput level; more specifically, our 4D-parallel neuromorphic SC-CNN can give nearly 100 times better efficiency in ADP (area-delay product) over 2D-parallel architectures. Fifth, our error injection experiments demonstrate that our SC-CNNs can be significantly more fault-tolerant than conventional digital implementations. These results suggest that our SC-CNN, which has several traits of neuromorphic computing, such as compact and efficient neurons, flexible precision, and high fault tolerance, can still be highly accurate and scalable similar to deep learning models.

The rest of the paper is organized as follows. After reviewing the related work in section 2, we present the SC-CNN and neuron-level optimizations in section 3. In section 4, we present the network-level optimizations including those targeting neuromorphic applications. In section 5, we present our experimental results, and section 6 concludes the paper with a summary of the work and future directions.

## 2. Related Work

### 2.1. SC-Based Neural Network

The most closely related to our work is SC-based neural networks. Thanks to low implementation cost and high error resilience, SC is seen as a promising approach to accelerating applications in certain domains including image processing and neural network. Previous work on SC-based acceleration of CNNs can be classified into two categories: fully parallel and tile-based.

In the fully parallel approach (Kim et al., 2016; Li et al., 2016, 2017a,b; Ren et al., 2016, 2017), all neurons are implemented spatially using dedicated hardware resources, and they operate in parallel such that the neural network circuit will produce output as the wave of input data sweeps through the circuit. It could have very high energy efficiency owing to the fact that it does not involve external memory access to store intermediate result, but has limited applicability because it cannot support arbitrarily large CNNs.

The tile-based approach (Sim and Lee, 2017; Sim et al., 2017), on the other hand, is more scalable in terms of the number of layers and neurons supported, since it works by tiling the computation of a layer into smaller fixed-sized arrays, each of which is performed by the same hardware block. This is also the approach employed by all recent hardware accelerators for CNNs (Chen et al., 2014, 2017). The intermediate results are saved to and reloaded from buffers, which are typically on-chip and backed by external memories. Now for efficiency reasons in terms of storage and external memory bandwidth, SC data should be saved in memories as conventional digital numbers, in which case every on-chip memory access would require a conversion between SC and digital representations, which is a considerable overhead in the tile-based approach.

A recent technique (Sim and Lee, 2017) proposes a new SC-MAC (multiply and accumulate) algorithm by combining the SNG (stochastic number generator), SC multiply operation, and an addition in the digital domain. This input and output of the SC-MAC is conventional digital. As such, it fits nicely with the tile-based approach, greatly reducing the conversion overhead. However, the inherent precision disadvantage of SC—that SC requires exponentially longer bitstreams as precision increases—has so far kept SC from being competitive on larger CNNs, such as AlexNet (Krizhevsky et al., 2012).

In terms of accuracy, both the fully parallel (Kim et al., 2016; Yu et al., 2017) and tile-based approaches (Sim and Lee, 2017; Sim et al., 2017) have shown competitive result against conventional digital designs for small CNNs with about 10 classification categories, such as MNIST (LeCun et al., 2010) and CIFAR-10 (Krizhevsky and Hinton, 2009). It seems that the retraining capability of CNNs helps cope with the approximating nature of stochastic computing.

### 2.2. Neuromorphic Computing

Neuromorphic computing is multi-faceted. On the one hand, there is the neuromorphic engineering approach (Mead, 1990), where researchers try to design useful systems based on the elementary operations of devices, which could lead to much more efficient systems than conventional digital designs. On the other hand, many neuromorphic models (Izhikevich, 2003) and systems (Markram, 2012) aim to imitate or emulate the nervous system as faithfully as possible, which can aid, e.g., with brain scientists studying the nervous system. Some of the effort has led to the design of dedicated hardware chips, such as TrueNorth (Akopyan et al., 2015) and Loihi (Davies et al., 2018).

Due to the similarity between neuromorphic computing and deep learning, there is a hope that the neuromorphic approach can 1 day lead to a much better neuron model than what is used today. For instance, spiking neuron, such as leaky integrate-and-fire (LIF) model (Maass, 1997) is referred to as the third-generation neuron model after McCulloch-Pitts neuron (McCulloch and Pitts, 1943) and perceptron (Hornik et al., 1989). Since spiking neurons generally use *timing* information, they may be able to achieve sparse, event-driven neural networks with much higher energy efficiency than perceptron-based neural networks used in today's deep learning (Roy et al., 2019). However, it remains to be seen whether SNNs can indeed show competitive performance as deep learning models on large datasets. To improve the efficiency of SNNs, a recent technique (Stöckl and Maass, 2020) reduces the number of spikes a neuron has to emit to pass information to subsequent neurons, by essentially encoding the information as a binary number where each bit position has a different weight. However, it discusses no hardware implementation, thus with no area- or energy-efficiency result. To improve the accuracy of SNNs, which is another way to improve efficiency, a training method (Wu et al., 2019) was proposed that uses an auxiliary artificial neural network that approximates the behavior of the coupled SNN and thereby enables back-propagation-based training for the SNN. Our training method is also based on back-propagation, which is why our models can show very high accuracy. Note that the training method, such as Wu et al. (2019) does not eliminate the need for (re)training for SNNs, but it only makes SNN training converge better and faster.

### 2.3. Deep Learning Hardware

Recent CNN hardware implementations (Chen et al., 2014, 2017; Han et al., 2016) all have their precision fixed at design time. This has an obvious disadvantage when the application's precision requirement is different from the designed precision, that is, accuracy loss (when the needed precision is higher) and efficiency loss (when it is lower). This mismatch can happen even within a single CNN, such as varying precision requirement among different layers (Judd et al., 2016), which causes some inefficiency even when the accelerator is running the CNN for which it is designed. One solution to the precision mismatch problem is to use bit-serial hardware, such as bit-serial multiplier (Judd et al., 2016). Our solution can provide an alternative solution, which has other advantages, such as error resiliency (see section 5.7) as well as being more efficient than the bit-serial approach (see section 5.4).

In comparison, SC can be more efficient partially owing to the optimizations proposed in this paper. This helps close the efficiency gap between SC and bit-parallel conventional digital for a wider range of precisions as we show later in Figure 7, while still retaining the benefits of SC, such as DPS. Also the effect of DPS could be higher in SC than in conventional digital, since 1-bit reduction in SC may reduce the delay of computation by about 50% as shown in Figure 1. In the figure, the *y*-axis shows computation delay, which is given by the bitstream length needed to deliver the precision on the *x*-axis. The exponential relationship holds for both the conventional SC (Kim et al., 2016) and the new SC-MAC (Sim and Lee, 2017).

## 3. Dynamic Precision Scaling SC-CNN

Our neuron model is built on top of SC-MAC (Sim and Lee, 2017), which uses a deterministic and optimized SC multiply algorithm. We first briefly review it and its application to neural networks, and present two extensions: (i) DPS optimization that allows the hardware precision to vary dynamically with little overhead, and (ii) HRS optimization that can be dynamically enabled.

### 3.1. Analysis of Baseline SC-MAC

**Figure 2:**
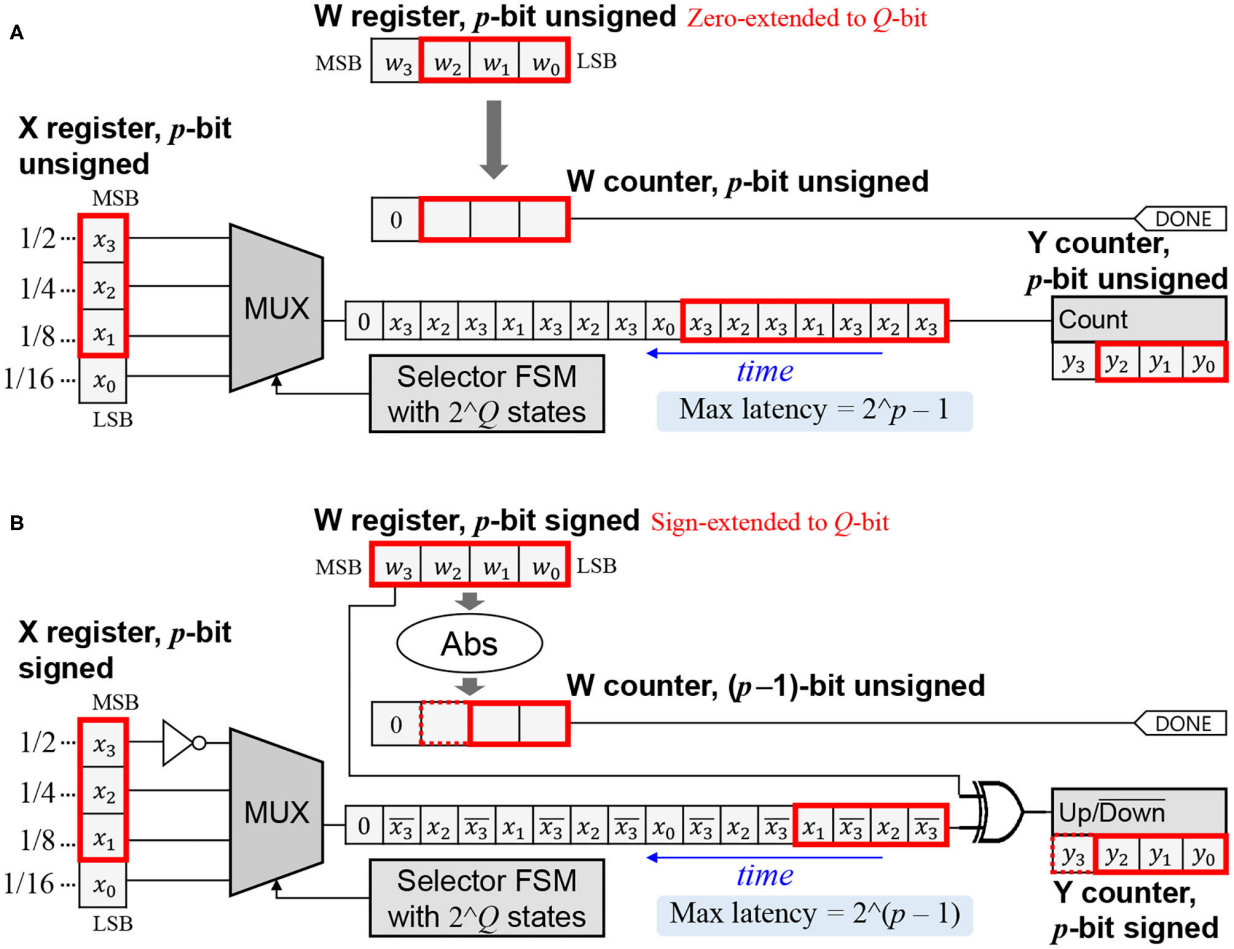
SC-MAC from Sim and Lee (2017) extended for dynamic precision. Datapath remains the same; only the control is changed (*p*: dynamic precision, *Q*: the maximum supported precision). **(A)** Unsigned version. **(B)** Signed version.

The key features of the baseline SC-MAC (Sim and Lee, 2017) include SNG integration, a novel SC multiply algorithm, and variable latency, which help it achieve superior efficiency as compared with conventional SC-based MACs, such as Kim et al. (2016). The MAC unit takes two operands labeled *x* and *w*, and generates output *y* that should approximate *xw*. All the inputs/output are represented as conventional digital, as illustrated in Figure 2 (shown in red is for dynamic precision discussed in the next section).

Let us first consider the unsigned version, where the inputs/output are interpreted as fractional numbers between 0 and 1. Let integer *X* = *x*2^*Q*^ and integer *W* = *w*2^*Q*^, where *Q* is the width of the X and W registers. The MUX-FSM circuit is designed to generate a bitstream whose signal probability is close to *x* (Sim and Lee, 2017). Thus, counting bits from the *x*-bitstream for *W* cycles gives approximately *xW* = *xw*2^*Q*^. Therefore, *y* ≈ *xw* with *Q*-bit precision.

In the signed version, the inputs/output are 2's complement numbers between −1 and 1. Since the MSB (most significant bit) is used as the sign bit, *X* = *x*2^(*Q*−1)^ and *W* = *w*2^(*Q*−1)^. The W counter is initialized to the absolute value of *W*. If *W* is positive, feeding the *x*-bitstream to the Y counter (which is now an up/down counter) for *W* cycles will give approximately *xW* = *xw*2^(*Q*−1)^. To understand why, note (i) unsigned interpretation of X register after inverting the MSB is (*X* + 2^(*Q*−1)^)/2^*Q*^, and (ii) the expected contribution of a single bit *z* to an up/down counter is (2*z* − 1). Thus, the expected change of *Y* per cycle is 2(*X* + 2^(*Q*−1)^)/2^*Q*^ − 1 = *x*. If *W* is negative, the *x*-bitstream is inverted, resulting in the negated value of *x*(−*W*), or *xW* in the Y counter. In either case, *y* ≈ *xw* with *Q*-bit precision (including the sign bit).

### 3.2. Acceleration of Neural Network

**Figure 3:**
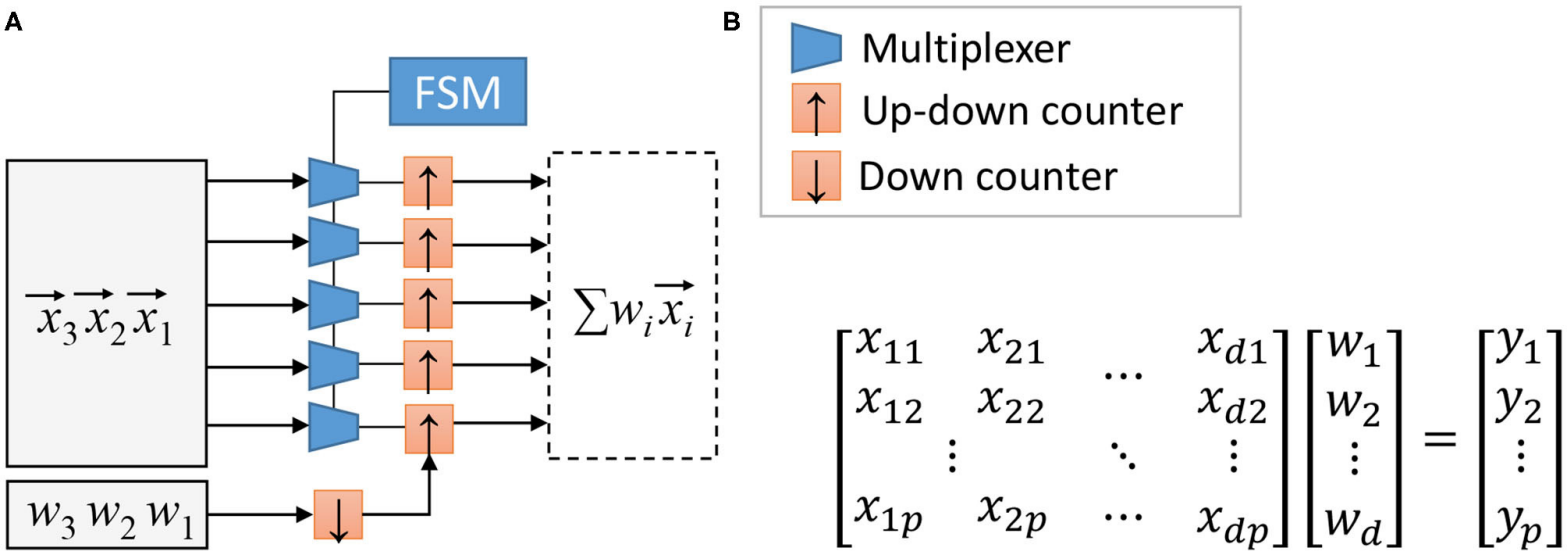
**(A)** Stochastic computing (SC) matrix-vector multiplier (simplified) and **(B)** the operation it performs, where $y_{j}=\underset{i}{\sum }w_{i}x_{ij}$, reproduced from Sim and Lee (2017).

The baseline SC-MAC can be used to accelerate convolution and linear (also known as fully connected) layers (Sim and Lee, 2019). The first question is how to combine multiple MACs to create an array of MACs. Figure 3 illustrates the matrix-vector multiplier (MVM) block proposed in Sim and Lee (2017), which is to share the weight parameter (*w*) among the MACs. The *j*th up/down counter in Figure 3 accumulates the product terms *x*_*ij*_*w*_*i*_, eventually computing $\underset{i}{\sum }x_{ij}w_{i}$. In this sense, one SC-MAC, which consists of an MUX and an up/down counter, can be regarded as a synapse-neuron pair.

Constructing the MAC array in this manner has two important benefits. First, since the latency of the SC-MAC is dependent on *w*, this scheme ensures that all the SC-MACs in an MVM block finishes simultaneously; in other words, there is no synchronization overhead within an MAC array. Second, the FSM and the down counter connected to *w* can be shared across all MACs within an MVM block, leading to cost-efficient hardware.

To use the MVM array as the main computation engine of a neural network accelerator, we also need to decide the dataflow, or how to parallelize computation. For convolution layers, for instance, computation of the output feature map (OFM) uses the same weight parameters along the width and height dimensions. Hence the convolution computation can be parallelized along the two dimensions as suggested in Sim and Lee (2017), which corresponds to output stationary according to the taxonomy of Chen et al. (2017). To provide the input to the 2D MAC array one can use the input reuse network (Rahman et al., 2016) between the input feature map buffer and the MAC array, or rearrange input data in advance with some duplication as is typically known as im2col (Chetlur et al., 2014). We later extend the 2D parallelism to make a better use of hardware area for neuromorphic applications (see section 4.6).

### 3.3. Dynamic Precision Scaling Extension

The extension to support DPS on the SC-MAC costs very little hardware. In fact, the datapath remains almost the same except for a few gates, the major difference being in the control logic. Here, we explain the operation of DPS SC-MAC.

The DPS SC-MAC has an additional input *p*, which is the precision of the input/output values. Figure 2 illustrates an example with *p* = 3 where the maximum precision *Q* is 4-bit.

Let us first consider the unsigned version, where the operands and the output are interpreted as unsigned numbers between 0 and 1. The output, being accumulated, may grow larger than 1, for which we add extra bits in the Y counter. The X register holds the integer version of *x*, or *x*2^*p*^, aligned at the MSB. The remaining bits, if any, are not used. The W register is initialized to the integer version of *w*, or *w*2^*p*^, zero-extended to fill the *Q*-bit register, i.e., *W* = *w*2^*p*^. The selector FSM is unchanged regardless of *p*.

It is easy to see that the latency of multiplication is *W* = *w*2^*p*^ cycles, which is at most 2^*p*^ − 1. During this period, the LSB (least significant bit) part of X register that is not initialized from *x* is unused (*x*_0_ in the example). Thus, counting the *x*-bitstream still approximates *xW*, with less accuracy due to the reduced precision of *W*. Since *Y* ≈ *xW* = *xw*2^*p*^, *y* ≈ *xw* with *p*-bit precision.

In the signed version, the X register holds *x*2^(*p*−1)^ aligned at MSB. After inverting the MSB, the unsigned interpretation of X register is 0.5+*x*/2 with *p*-bit precision, assuming a decimal point right before MSB. The W register is initialized to *w*2^(*p*−1)^, sign-extended to *Q*-bit, i.e., *W* = *w*2^(*p*−1)^. If *W* is positive, the Y counter holds *xW* = *xw*2^(*p*−1)^ after *W* cycles. If *W* is negative, it holds −*x*(−*W*) = *xW* due to the XOR gate. In either case, *y* ≈ *xw* with *p*-bit precision including the sign bit.

The latency of signed multiplication is |*w*|2^(*p*−1)^ cycles. The maximum latency of DPS SC-MAC is 2^(*p*−1)^ when *w* = −1, in which case the W counter requires *p*-bit, as indicated by the dotted box in Figure 2B (but it never needs more than *Q* bits). Similarly, the Y counter needs *p*+1 bits, which may exceed *Q* bits; however, the Y counter has extra bits already in order to serve as an accumulator.

Since the *p*-bit precision of *y* always starts from LSB, it means that the decimal point will have to move depending on the precision. Fixing the decimal point can be done with a single shifter.

### 3.4. Half-Range Specialization

Since the latency of an SC-MAC is exponential to the precision, saving even 1 bit is very worthwhile. HRS is based on the observation that the range of certain variables, namely, input activations, are guaranteed to be non-negative due to the particular shape of activation function (i.e., ReLU) used in the preceding layer.

One way to exploit the limited range of input is through a data scaling framework as in section 4.3. But data scaling works best for symmetrical ranges, and making asymmetrical ranges symmetrical incurs additional overhead.

Alternatively we can make a full use of input precision in our DPS SC-MAC of Figure 2B by treating *x* as unsigned. This effectively increases *x*'s precision to *p*-bit while the precision of *w* remains the same [i.e., (*p* − 1)-bit due to 1-bit sign]. Since *w* is unaffected, latency is also the same. The main effect of this scheme is accuracy improvement: in our evaluation, (*p* − 1)-bit multiplication with HRS shows a similar accuracy as *p*-bit multiplication without HRS (see Figure 10). Conversely, HRS can achieve a similar accuracy with 1-bit less precision, or at half the latency.

It is important to note that HRS cannot guarantee the same accuracy as that of 1-bit lower precision, since *w*'s precision is not increased. However, input activation often turns out to require a higher precision than weight parameters Judd et al. (2015), which explains why increasing *x*'s precision through HRS is very effective in practice. On the other hand, if a layer or network requires higher-precision weight (*w*) than input (*x*), the HRS advantage of cost-free increase of *x* precision by 1 bit will not be very useful, since weight precision is the bottleneck and determines the SC-MAC's precision.

**Figure 4:**
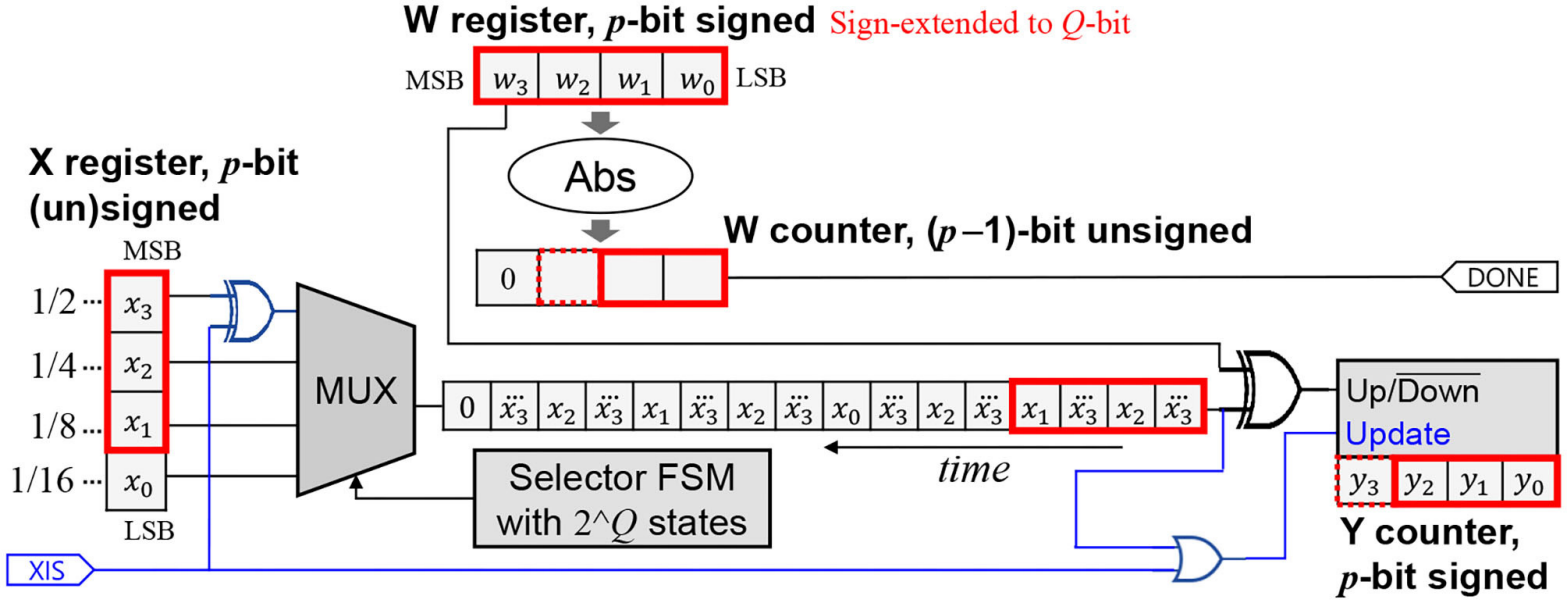
Supporting HRS mode for *x* along with the normal mode. Extension is shown in blue. The new input XIS indicates whether *x* is signed.

Also important to note is that in order to support layers whose input is not necessarily one-sided (e.g., the first layer), the hardware must retain the original behavior of Figure 2B. Thus, we make HRS runtime-programmable through an extra input XIS (meaning “*x* is signed”), as shown in Figure 4.

When XIS is 1, the hardware degenerates into the signed version. When XIS is 0, the *x* part becomes like the unsigned version, and the up/down operation of the Y counter is suppressed if the *x*-bitstream's output is 0, essentially making it perform either up or down depending on the sign of *w*, which ensures a correct operation.

## 4. Design Optimizations for DPS SC-CNN

### 4.1. Hardware Precision vs. Software Precision

So far our notion of precision has been the width of a variable in the application program as represented in conventional digital, which typically ranges up to 32-bit. Quantization is to reduce the precision in the application code. Thus, this kind of precision may be called *software precision*. When converted into SC, a variable of *n*-bit software precision requires about a 2^*n*^-bit bitstream.

While a bit-serial multiplier as in (Judd et al., 2016) computes only one bit at a time, it is quite common in SC to employ bit-parallel hardware due to the relative simplicity of an SC multiplier compared to the rest of SC-MAC. To generate a variable of *n*-bit software precision, a bit-serial SC multiplier needs 2^*n*^ cycles, but a *k*-bit parallel SC multiplier can do it in 2^*n*^/*k* cycles. We define *hardware precision* as the base-2 logarithm of *k*.

It is straightforward to extend the DPS SC-MAC to a bit-parallel version, which supports integer hardware precisions for efficiency reasons. It is based on the bit-parallel version of the baseline SC-MAC (Sim and Lee, 2017).

### 4.2. Design Flow

A design objective is to minimize ADP while meeting accuracy constraint, which we set to be 1% point below the reference accuracy achieved by an unquantized version (i.e., floating-point implementation). We consider the following design parameters: (i) data scaling parameters, (ii) software precision of each layer, and (iii) hardware precision of SC-MAC. Next we discuss each of these.

### 4.3. Determining Data Scaling Parameters

Previous work (Lin et al., 2016) has pointed out the importance of scaling input data to better utilize the limited range of SC or fixed-point representations. The idea is to scale more than covering the worst case input data, such that some of the input values go out of range. It may introduce errors to some input, but those in the range can be represented more precisely. More-than-worst-case scaling is particularly effective when the out-of-range input data get saturated. To avoid the overhead due to scaling, scaling parameters are typically restricted to powers of 2.

The issue here is how to determine data scaling parameters, the effect of which seems highly unpredictable. We use the following scheme.

1. Determine the scaling factor so that all values are within range (i.e., worst-case design).
2. Double the scaling factor and check whether the recognition accuracy improves.
3. Repeat the above while there is improvement.

The above procedure is repeated for each layer, starting from the first layer. We do not retrain the CNN during this procedure. We find this scheme robust as it does not rely on any arbitrary design parameter, which is a major advantage of the scheme. While this algorithm is greedy and not able to address the possible inter-dependence issue among layers, doing so would run into a combinatorial problem, which may require a prohibitive amount of resources for large CNNs.

### 4.4. Determining Software Precision of Each Layer

Similar to data scaling parameter exploration, here we optimize one layer at a time in order to avoid combinatorial problems. There are also differences. First, precision optimization uses retraining, which is crucial to get meaningful accuracy at low precisions. On the other hand, retraining takes much longer than inference, and can take hours and days for the SC version even when using GP-GPUs for simulation. Second, higher precision is more detrimental than a lower precision can save. Thus, we first find the *uniform* precision for the SC version that satisfies the accuracy constraint with retraining. This can be solved in linear time, since all layers have the same precision. The uniform precision is used as the precision upper-bound for each layer. Third, knowing the uniform precision also helps determine hardware precision (see the next section). Fourth, to speed up the search we use the result of conventional digital implementation's optimized precision. However, since there is usually a gap between the precisions of the two, we use a concept called *precision slack*.

To illustrate *precision slack*, suppose that a conventional digital implementation is optimized to have the following precisions across five layers: 10–9–5–6–8. Precision slack is the difference in precision between the highest and the current layer, e.g., 0–1–5–4–2 in this example. Then we subtract precision slack from the uniform precision value to get the precision lower-bound. The rationale is that while the SC version gives higher reward for lower precision, its accuracy is also more sensitive to it. Also using very low precisions gives diminishing return (see Figure 1) while often hurting accuracy too much. Having a lower-bound makes it easy to do binary search instead of linear search, saving retraining time.

### 4.5. Determining Hardware Precision

Hardware precision affects both delay and area of our SC-CNN (see section 5.3). Therefore, the decision in this step could affect the optimality of the precision setting found in the previous step as ADP can change as we use a different hardware precision. To avoid this problem, we run this step twice, first for the uniform precision value, then after non-uniform precision setting is found. Finding the best hardware precision is straightforward, and can be done quickly as it has only a linear complexity and does not require retraining (changing hardware precision does not affect recognition accuracy, but only ADP).

### 4.6. Neuromorphic Optimizations

#### 4.6.1. Motivation

One key difference between neuromorphic vs. deep learning hardware is the separation of computation and memory. In the nervous system, memory is distributed and computation is tightly integrated with memory, whereas in today's deep learning hardware, memory is clearly separated from the compute engine, which can create performance bottleneck for certain applications due to memory wall.

The main reason why today's deep learning architectures use the von Naumann architecture is efficiency. Because digital MACs are large, a typical chip can have only so many of them, which we must use iteratively, or in a time-multiplexed manner, in order to handle large layers and networks. Also because of the large granularity of individual MACs, it would be quite inefficient and difficult in terms of placement and routing if we distribute them among memory blocks.

Contrary to a digital MAC, which consists of an *n*-bit multiplier and an *m*-bit accumulator (*m* > *n*), an SC-MAC is extremely small. It allows us to build a massive array of neurons and synapses on a single chip, for which we explore a much more parallel architecture than the previous SC neural networks.

We also explore a tight integration of memory (e.g., SRAM blocks) with SC-MACs. Even though our SC-MAC takes digital numbers (X and W) as input, only one bit is used at a time (see Figure 4). By rearranging the bits in the memory, the MUX can be made redundant, with its function merged into the address decoder of an SRAM block.

#### 4.6.2. More Parallel Architecture for SC-CNN

**Figure 5:**
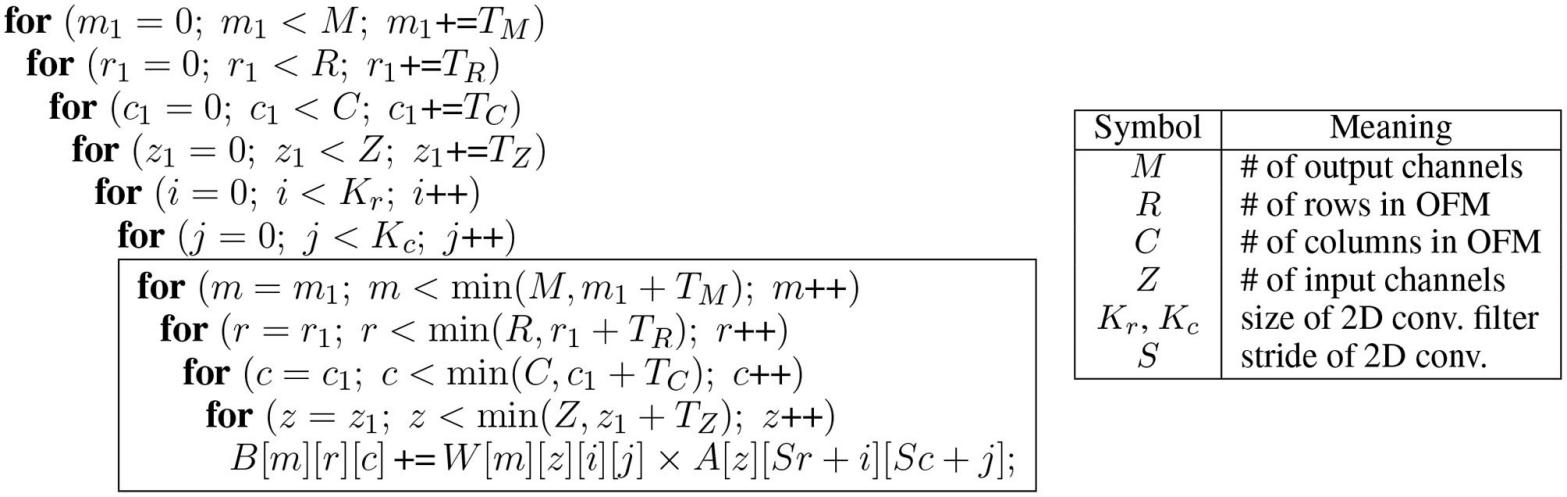
Convolution layer tiled along four loop levels (*M, R, C, Z*). Arrays *A*, *B*, and *W* are input feature map (IFM), output feature map (OFM), and weight parameters, respectively. The four innermost loops are hardware-unrolled, creating a 4D-parallel architecture.

While the size of our SC-MAC depends on the hardware precision as explained in section 4.1, it can be up to 63.9 times smaller than a digital MAC. To better utilize the massive number of SC-MACs available, we parallelize along all dimensions of the convolution kernel. Figure 5 lists the C code of a convolution kernel that is tiled along all four dimensions of *Z, M, R, C*. The remaining two loop levels not tiled, *K*_*r*_, *K*_*c*_, are typically very small. Those tiled loops are unrolled in hardware, meaning that the MAC array consists of *T*_*Z*_ × *T*_*M*_ × *T*_*R*_ × *T*_*C*_ SC-MACs, and is able to perform the same number of SC-MAC operations every cycle. This is in contrast with the previous SC-CNNs (Sim and Lee, 2017; Sim et al., 2018), where only *R, C* dimensions are unrolled, thus having saturating efficiency when the array size increases (see section 5.6). Conventional digital accelerators also, such as Chen et al. (2014, 2017), not having been optimized for such a high degree of parallelism, suffers the same limited efficiency issue.

Parallelizing along the *M*-loop is similar to parallelizing along the *R*- or *C*-loop because *M*, *R*, and *C* are all output feature map dimensions; we replicate the 2D MAC array *T*_*M*_ times. But there is a downside. Each of the 2D MAC arrays uses one weight value per cycle, hence there are *T*_*M*_ weights overall that need to be supplied per cycle. Consequently, the *T*_*M*_ MAC arrays may have different latency values, which can incur synchronization overhead among the 2D MAC arrays.

The main idea of parallelizing along the *Z*-loop is to increase the number of inputs for the up/down counter in Figure 4 by *T*_*Z*_ times. Instead of having a single bitstream, we now have *T*_*Z*_ bitstreams coming from different input channels; thus, we employ a *T*_*Z*_ bitcount logic to combine *T*_*Z*_ bits into an integer, which is then accumulated. In fact, the up/down counter in Figure 4 has three operations, i.e., up, down, and no-op (when update is zero), due to the HRS optimization. Hence, the input bitstreams are ternary, and the bitcount logic is extended to handle ternary input. Similar to the parallelization along the *M*-loop, the weight parameters from the *T*_*Z*_ input channels may all be different. There are *T*_*Z*_ down counters corresponding to the *T*_*Z*_ weights. The done signal from a down counter forces the corresponding ternary input to zero, which makes the input effectively ignored by the bitcount logic.

#### 4.6.3. Tight Integration of SRAM and SC-MAC

Among the main components of an SC-MAC, i.e., MUX and up/down counter, the MUX can be made redundant if we rearrange the input data in the SRAM. Suppose input *x* is 8-bit. In the conventional memory storage, each byte of the input SRAM contains one value of *x*, the next byte containing the next *x*, and so on. In the previous work (Sim and Lee, 2017; Sim et al., 2018), all these values of *x* are loaded simultaneously into the input registers, but only one bit is accessed per cycle through the selector FSM.

More specifically, let *x*_1_, *x*_2_, etc. be 8-bit values loaded to the input registers of an MAC array. In other words, *x*_*j*_ is the *j*th element of the input vector ${\vec{x}}_{i}$ in Figure 3A. Let $x_{j}^{\left(k\right)}$ be the bit *k* of *x*_*j*_ where 0 ≤ *k* ≤ 7. Then in the first cycle we need a set of bits, $x_{1}^{\left(7\right)}$, $x_{2}^{\left(7\right)}$, etc., and in the next cycle we need another set of bits, $x_{1}^{\left(6\right)}$, $x_{2}^{\left(6\right)}$, etc. This scheme may be called *bit-major*.

Now we propose to store the input data in such a way that the bits needed together are stored together in the same byte as much as possible. For instance in the above example, bits $x_{1}^{\left(7\right)},x_{2}^{\left(7\right)},\cdots \;,x_{8}^{\left(7\right)}$ will make up one byte, and $x_{1}^{\left(6\right)},x_{2}^{\left(6\right)},\cdots \;,x_{8}^{\left(6\right)}$ will make up another byte. The latter byte may never need to be accessed simultaneously as the former, hence can be stored in the depth direction of an SRAM block. We call this scheme *lane-major*. Using the lane-major scheme can eliminate both input MUXes and input registers. The scheme can also reduce the input memory bandwidth, or the maximum number of bytes we read from the input memory in a cycle, by *N* times compared with bit-major, where *N* is the width of input in bytes times 8. For instance, if the input data are 12-bit wide, it still requires 2 bytes, thus the saving is 16 times. Using the lane-major scheme does not significantly affect the capacity of the input memory needed but does affect the aspect ratio; we now need deeper memory.

On the other hand, since we have eliminated input registers, we must access the input SRAM every cycle, potentially increasing the energy consumption. In the worst case, an 8-bit input data may need to be accessed 256 times in the lane-major scheme. However, the actual number of accesses depends on the weight value, which is typically small. Also when using dynamic precision scaling, the actual precision at the moment can be much less than the width of the input data stored in the memory. Thus, our scheme fits well with DPS.

While the input data are stored as lane-major, the output data as produced in an SC-MAC follows bit-major. Therefore, we need to convert the bit packing scheme of the output data. This can be done when the output data are loaded from the external memory to the on-chip input buffer, at which time we also apply the im2col transformation (Chetlur et al., 2014). In addition, we can optimize away the XOR gate required by HRS, by doing the MSB flipping in advance. It can be done during the bit packing conversion.

## 5. Experiments

### 5.1. Experimental Setup

To evaluate our approach, we use CNNs targeting ILSVRC2012 (ImageNet Large Scale Visual Recognition Challenge 2012), such as AlexNet, VGG, and GoogLeNet, in addition to smaller ones. For training and accuracy evaluation, we use Caffe (Jia et al., 2014) extended to model the functionality of DPS SC-MAC. For retraining (also called fine-tuning), 5,000 update iterations were performed starting from the reference models of the Caffe Model Zoo.^2^ For learning parameters, such as weight decay and batch size, we use the same values as used in the reference solver script provided with the model, with the only exception of the base learning rate, which is scaled down by 10× from that of the reference script. During retraining, forward propagation is done using the SC algorithm but back-propagation is done using floating-point arithmetic with weight update done to real-valued weights, which is essentially the same procedure used in training quantized neural networks (Hubara et al., 2017). Note that retraining is needed not only for SC-DNNs but digital DNNs also, and that the computational overhead of retraining is very little compared with that of the baseline training (5,000 vs. 450,000 iterations in the case of AlexNet). Recognition accuracy is reported for the first 10,000 images (out of 50,000) of the ImageNet validation set. The SC-CNN architecture is modeled cycle-accurately to generate exact cycle counts in a data-dependent manner.

We have extended the SC-MVM (matrix-vector multiplier) (Sim and Lee, 2017) to support our DPS SC-MACs, which is referred to as DPS SC-MVM. Our DPS SC-MACs have a few variants depending on hardware precision. Our DPS-2^*p* processes 2^*p*^ bits per cycle, therefore being roughly equivalent to *p*-bit parallel digital logic. We have implemented the previous SC-MVM (Sim and Lee, 2017), our DPS SC-MVM, and the conventional digital baseline MVM in Verilog and synthesized them using Synopsys Design Compiler. All syntheses were done for the same target frequency of 1 GHz, although SC is likely to meet higher frequency. The conventional digital baseline uses fixed-point binary multipliers with rounding accumulators. The area for Stripe (Judd et al., 2016) is estimated to be 207% of the digital baseline as per the paper, which however does not provide power result. Only convolution layers are accelerated in all the approaches compared, permitting us to use ideal convolution layer speedup for delay comparison. Maximal accuracy degradation is set to 1% point.

Our main figure of merit is area-delay product (ADP), which is the product of MVM area and average MAC cycles (thus the lower, the better), or its inverse representing operations per area. ADP is chosen because in addition to being a meaningful area-efficiency metric, it permits direct comparisons with previous work (e.g., Judd et al., 2016). Though we also report power and energy results for our designs, these metrics tend to vary a lot, affected more by such factors as on-chip and off-chip memory accesses (Chen et al., 2014), which are not the main focus of this paper, than the design of processing element (PE) arrays.

### 5.2. Area Overhead of our DPS SC-CNN

**Figure 6:**
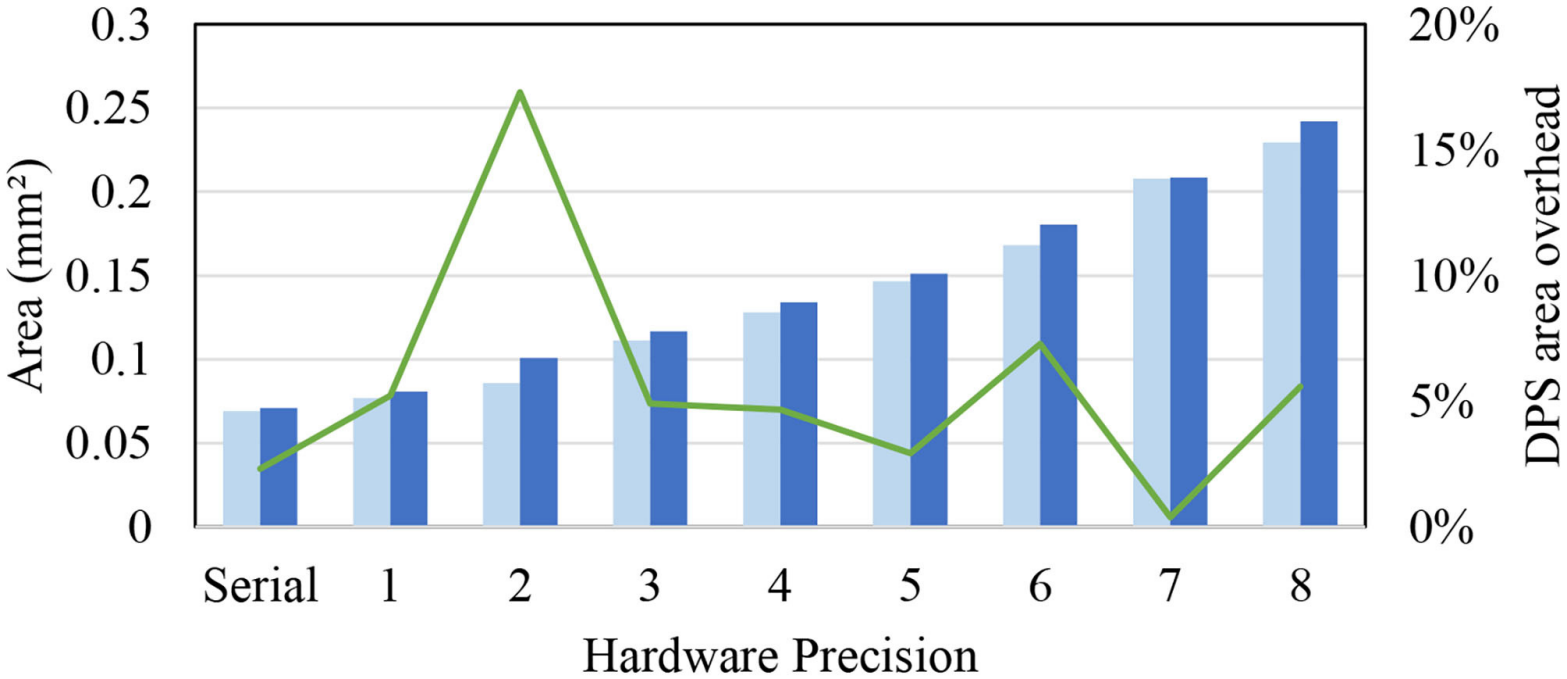
Area overhead of dynamic precision scaling (DPS) SC-MVM vs. SC-MVM.

Figure 6 compares the area of our proposed DPS SC-MVM against the previous SC-MVM (Sim and Lee, 2017). The DPS SC-MVM includes our optimizations, such as HRS, which have very small extra logic (see Figure 4). Not surprisingly, the graph shows that the area overhead of ours is mostly small, typically at around 5%, though varied depending on the hardware precision shown on the *x*-axis. The graph also shows that the area is linearly proportional to the hardware precision, or logarithmically to bit-parallelism. This is due to the optimization exploiting the structure of the bitstream ordering. Overall the average area overhead is 6%, which is small.

### 5.3. Effect of Software and Hardware Precision on ADP

**Figure 7:**
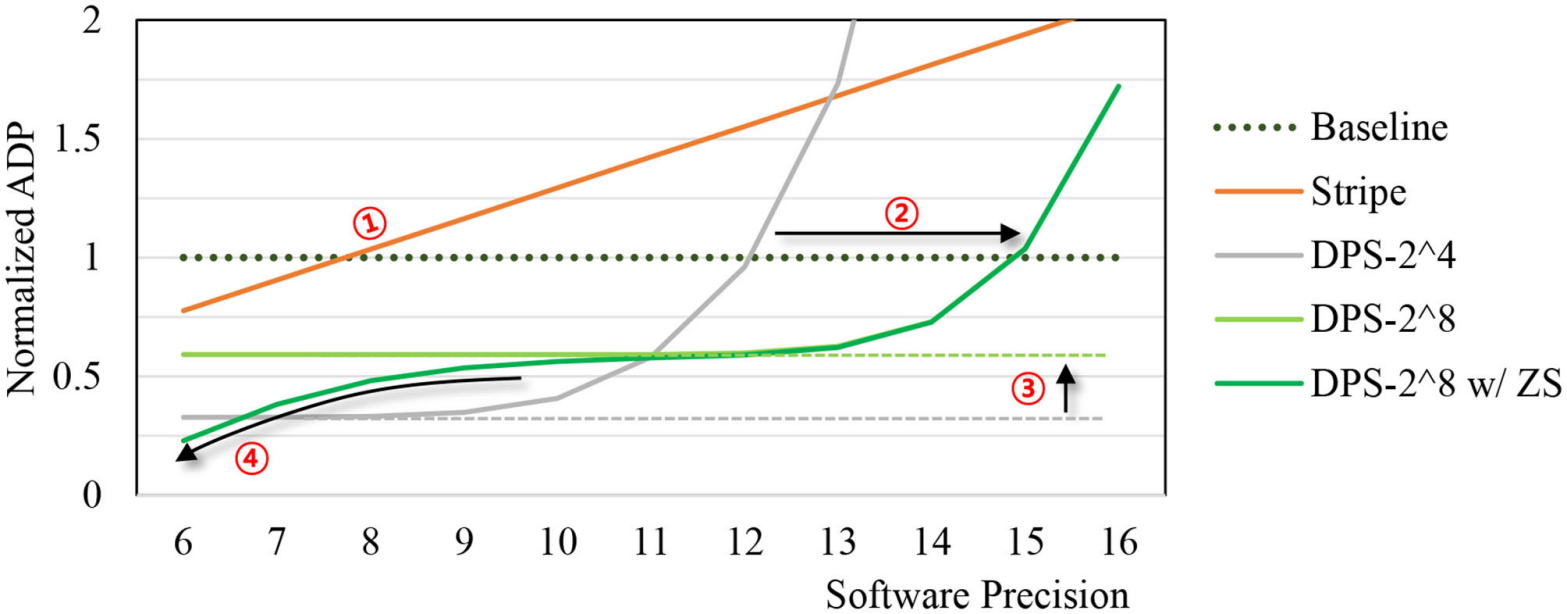
Area-delay product (ADP) vs. software precision.

Figure 7 shows the ADP trend as we vary software precision. For our ADP result, we use AlexNet parameters as our SC-MVM has data-dependent variable latency. The digital baseline does not support dynamic precision, thus has constant ADP. For Stripe, delay is proportional to the precision, resulting in linear ADP. The graph shows that Stripe becomes inefficient over the conventional digital baseline beyond 7- or 8-bit (➀). DPS-2^4, which is our DPS SC-MVM with hardware precision of 4, shows exponentially increasing ADP as software precision increases. But the range of software precision for which DPS-2^4 is more efficient than conventional digital is wider than that of Stripe.

DPS-2^8, which is our DPS SC-MAC with hardware precision of 8, can widen the efficient operating range even further (➁). At the same time, it has higher ADP than DPS-2^4 when software precision is lower (➂), as it is more optimized for higher precision workload. Some of the efficiency loss can be reclaimed by zero skipping (➃), which is to skip computation of multiplication whose weight operand is 0 (after quantization) as shown in the graph.

**Figure 8:**
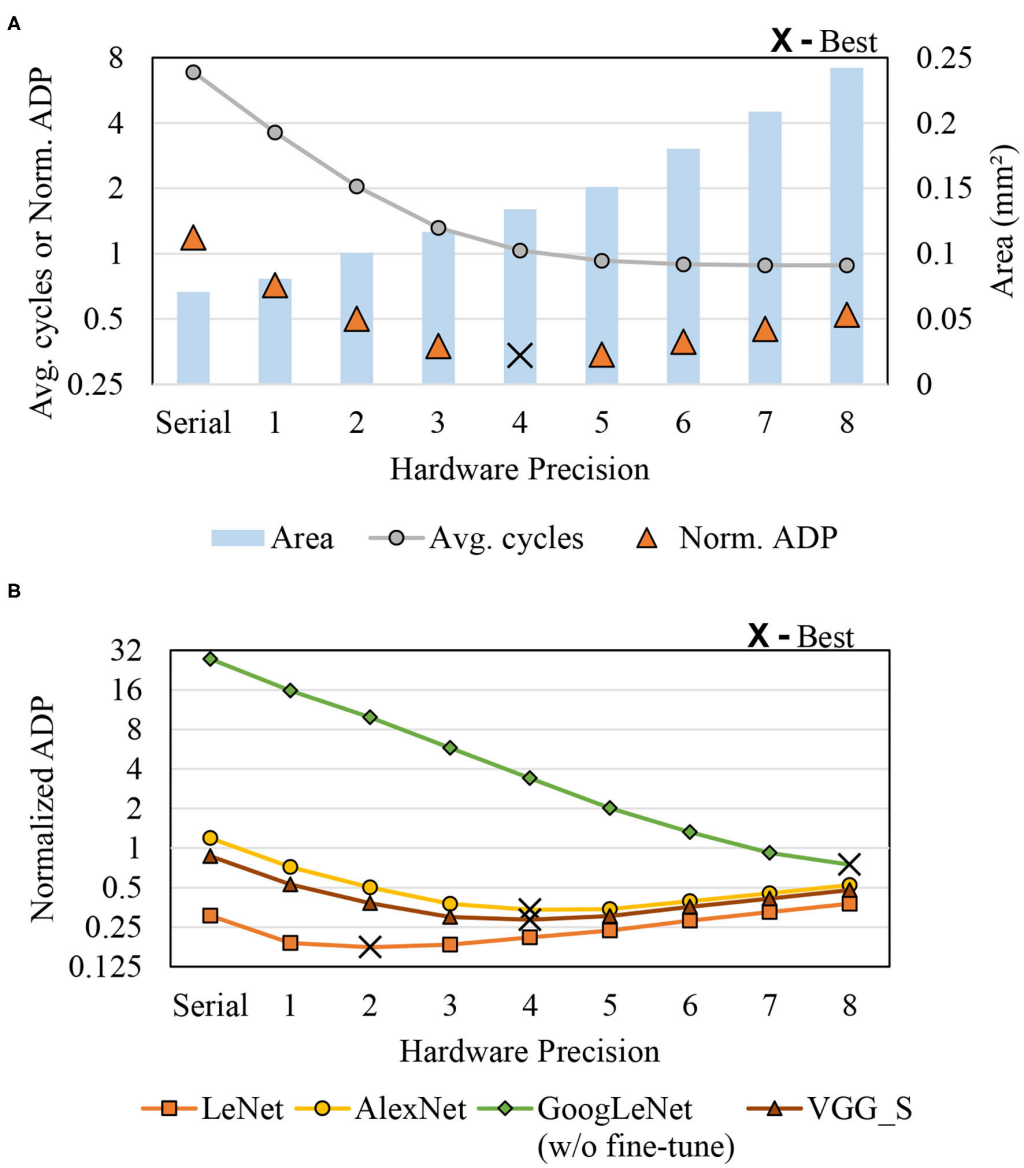
Area-delay product (ADP) vs. hardware precision. **(A)** AlexNet and **(B)** Multi-application.

As demonstrated previously, ADP depends on hardware precision. Figure 8 shows ADP vs. hardware precision (a) for a CNN (AlexNet) and (b) for multiple CNNs. As hardware precision increases, the average delay decreases until it reaches saturation, whereas area increases more or less linearly to hardware precision. This suggests that there is an optimal hardware precision to minimize ADP. In the case of AlexNet in Figure 8A, for instance, ADP is minimized at hardware precision of 4, or DPS-2^4. But in other CNNs, different points can be optimal as there are different weight distributions and precision requirements depending on the CNN. Figure 8B shows how ADP as well as optimal hardware precision changes depending on application. Understandably, large and complex CNNs seem to be better off with higher hardware precision. Our hardware precision for the multi-application scenario (see the next section) is chosen based on this profile.

### 5.4. Multi-Application Scenario

**Figure 9:**
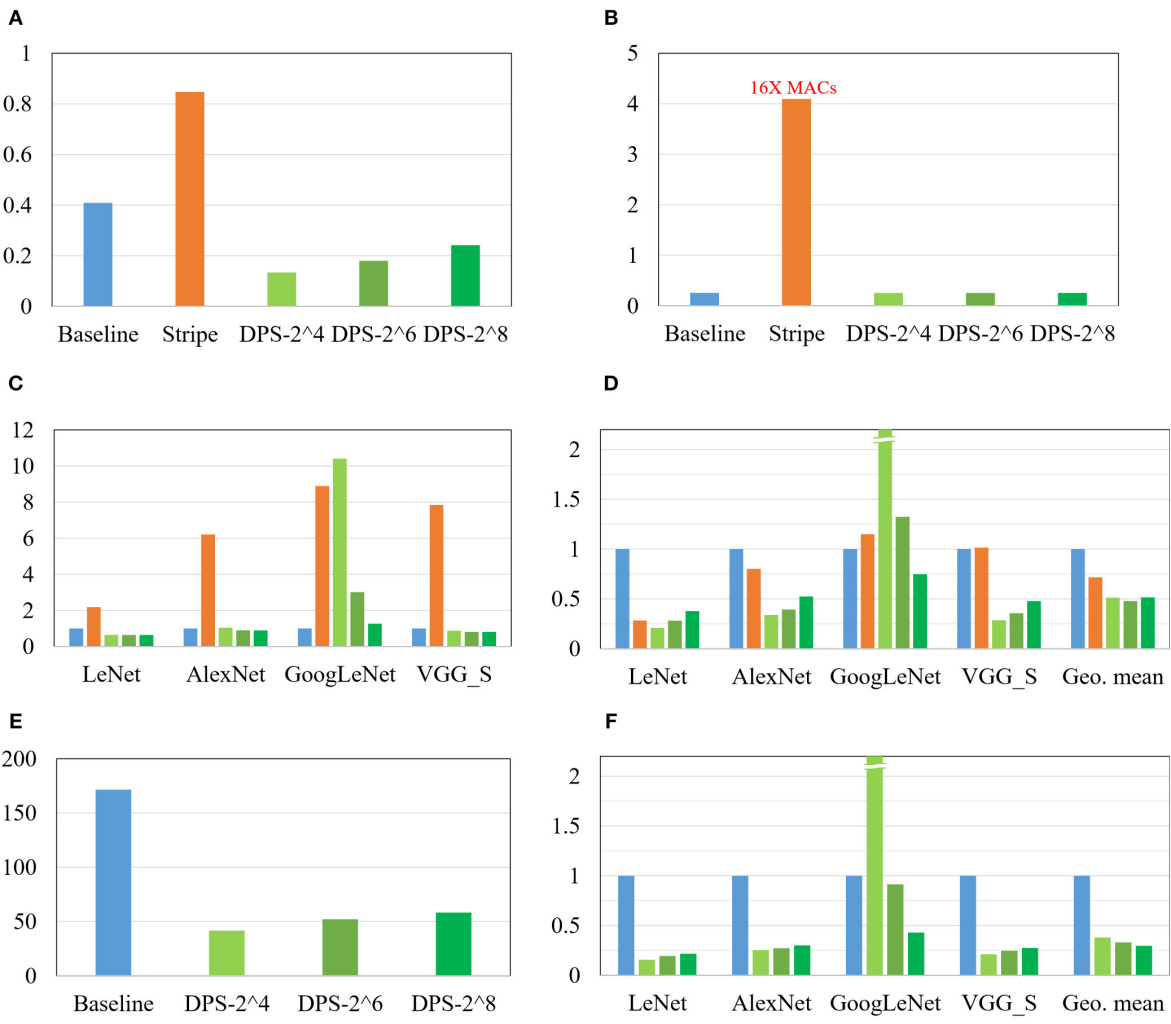
Comparison with the digital baseline and Stripe. **(A)** Area (mm^2^), **(B)** ^#^MAC of a tile (1 k), **(C)** Average cycles, **(D)** Normalized ADP, **(E)** Power (mW), and **(F)** Normalized energy.

Figure 9A compares our DPS SC-CNN and previous CNN implementations. First, ours is highly area efficient, which is not surprising given the area efficiency of SC. Since Stripe is bit-serial, we use 16 times as many MACs for Stripe as in the baseline, so that the two can have the same throughput in the worst case (i.e., when the CNN's software precision is 16-bit). Hence, Stripe has the largest tile area as shown in Figure 9B due to the large number of MACs it has; the others have 256 MACs only.

Figure 9C shows average MAC cycles and Figure 9D shows the ADP result, which is normalized to the digital baseline. First, all these results are from implementations that achieve <1% point accuracy drop from the reference floating-point implementations (see Table 1). That SC-CNNs can achieve this high accuracy for large CNNs is very significant. Also this is why this graph has no comparison with previous SC-CNNs. Second, at the same time the efficiency of ours as measured in ADP is actually higher than that of conventional digital, often significantly. For instance, DPS-2^8, which is optimized for large CNNs, shows consistently better results than the conventional digital designs. It also demonstrates the flexibility as well as efficiency of our DPS SC-CNN. Third, the optimal design as measured in geometric mean of ADP is DPS-2^6 for this mix of CNNs, which is obviously influenced by the existence of a small network. But our scheme can flexibly support different workloads through the hardware precision, while simultaneously being able to support dynamic software precision at runtime. Figures 9E,F present the power and energy comparisons, which show very similar trends as those of the area and ADP comparisons in the same figure. Overall, our DPS-2^6 can achieve over 2× and 1.5× improvements compared with the baseline and Strip, respectively, in terms of operations per area.

**Table 1:** Recognition accuracy (for 10K images) and dynamic precision scaling (DPS) precision setting.

**CNN**	**Baseline accuracy (float)**	**DPS accuracy**	**DPS precisions found**
MNIST	0.9904	0.9826	5 (uniform)
AlexNet (top-5)	0.8070	0.7999	10-9-8-9-9
GoogLeNet (top-5)	0.8926	0.8844	13 (uniform, w/o fine-tuning)
VGG_S (top-5)	0.8341	0.8247	9-9-10-9-10

### 5.5. Single Application Comparison

**Figure 10:**
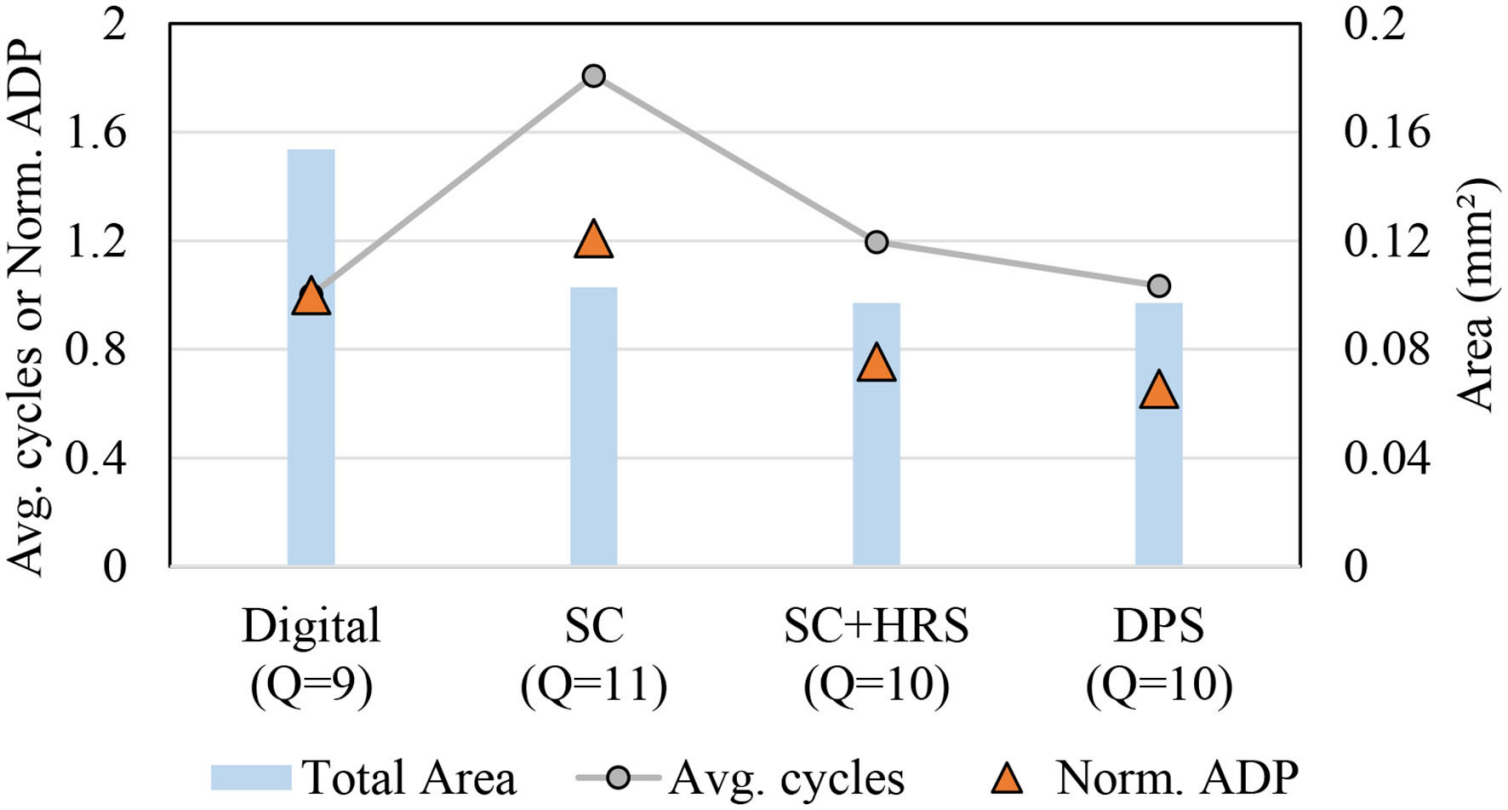
Comparison with previous SC-CNN on AlexNet.

We also compare different implementations including the previous state-of-the-art SC-CNN (Sim and Lee, 2017) for a single application scenario, i.e., when we design and use a chip for just a single CNN. We use AlexNet as the target CNN. Figure 10 shows area, average delay, and ADP results in one graph, all normalized to that of the digital baseline. For SC designs, hardware precision is set to 4. Maximum software precision supported (*Q*) is determined to be the minimum value that meets the recognition accuracy constraint, which is largely dependent on how accurate the MAC is. The digital baseline requires 9-bit while the previous SC-CNN requires 11-bit. Our DPS SC-CNN requires 10-bit with uniform precision; dynamic precision setting is listed in Table 1.

The graph shows that the previous SC-CNN has smaller area than the digital baseline but its average delay is much higher, which is attributed to the high precision requirement. Applying HRS to it (but not DPS) can reduce precision requirement by 1-bit with significant saving in delay, but its average delay is still higher than that of conventional digital. Applying DPS further gives 14% reduction in ADP, achieving the best efficiency. The relatively weak impact of DPS is due to the small number of layers in AlexNet and our limited precision exploration. For deeper networks and if we can use the optimal precision combination, the impact could be higher. Even with these limitations our proposed design achieves 34 and 46% reduction in ADP (or 52 and 85% increase in operations per area) over the digital baseline and the previous SC-CNN, respectively.

### 5.6. Efficiency of Neuromorphic Architecture

Thanks to its extremely small size, SC-MAC allows us to explore more flexible architectures including significantly more parallel architectures and tight integration of memories with compute elements. In one of those architectures, which more closely resembles the nervous system and therefore is referred to as *neuromorphic architecture*, we use the following parameters.

- It is bit-serial (hardware precision *p* = 0), meaning that each SC-MAC processes only one bit in a cycle.
- The tiling parameters (see Figure 5) are as follows: *T*_*Z*_ = *T*_*M*_ = *T*_*R*_ = *T*_*C*_ = 16.
- Input SRAM is directly integrated into SC-MAC as described in section 4.6.3.

The same synthesis setting is used as described in section 5.1 including the target clock frequency.

We compare three cases: digital, DPS SC-CNN (DPS-2^4), and the neuromorphic architecture. The result is summarized in Table 2. For fair comparison, we use the same number of synaptic connections, which is set to 64K (2^16^). This means that all the three architectures compared here have the same number of multipliers or their equivalents. In the table, the first two architectures, Digital and DPS-2^4, are the same as in Figure 9A, with only 256 MACs or synaptic connections, but added here for comparison. CNN cycles of MNIST for digital and DPS-2^4 are equivalent because the network precision requirement is 4 (excluding sign-bit) and DPS-2^4 can process the maximum length of stochastic stream at a cycle. Their “large” versions are created by increasing the tiling parameters; each has two tiling parameters, which are multiplied by 16 each. The ADP column is the geometric mean for both networks.

**Table 2:** Processing element (PE) array comparison.

	**Architecture (PEs)**	**#Synaptic connections**	**Area (mm2)**	**#Synaptic connections/Area**	**CNN cycles**		**Area-delay product (norm.)**	**Power (mW)**	**Energy (norm.)**
					**MNIST**	**ImageNet**			
Small	Digital	256	0.41	630	27.0K	3.8M	1	171.42	1
	DPS-2^∧^4	256	0.13	1,985	27.0K	4.3M	0.34	41.62	0.26
Large	Digital large^†^	64K	104.04	630	25.5K	2.3M	195.52	43884.54	195.52
	DPS-2^∧^4 large^†^	64K	33.02	1,985	25.5K	2.4M	62.73	10654.98	47.98
	Neuromorphic	64K	1.63	40,261	2.3K	1.1M	0.65	489.26	0.46
Small	DianNao*	256	0.85	302	41.6K	4.4M	2.79	132	1.03
	Eyeriss*	168	9.63	17	–	20.7M	–	–	–

Our neuromorphic PE array shows much higher synaptic density and better scalability than the scaled-up versions of Digital (the baseline) and our DPS-2^4. The Digital and DPS-2^4 PE arrays share the same architecture. For context, we also compare the baseline architecture with two previous accelerator architectures (^*^based on published papers, ^†^estimated).

The table suggests that among the three architectures with 64K synaptic connections, the neuromorphic architecture has the best area, performance, and ADP. In terms of area, the neuromorphic architecture shows several dozen times higher density, thanks to the use of bit-serial SC-MAC and input MUX elimination (enabled by SRAM integration). Yet, its latency is actually lower than that of the others. The DPS SC-CNN architecture suffers extremely low utilization, which is the main culprit of the architecture when the number of MACs is very high. Simply it is not designed to be very scalable, which is addressed in the neuromorphic architecture. While the neuromorphic architecture has its own weakness, i.e., synchronization overhead, it is relatively mild. Our simulation result shows that the overhead increases average MAC latency by about 2.96 and 6.97 times for MNIST and AlexNet, respectively, compared with when the synchronization overhead is ignored. As a result, the neuromorphic architecture achieves orders of magnitude improvement in ADP over DPS SC-CNN.

The table also provides comparisons with previous digital DNN accelerators. DianNao (Chen et al., 2014) is similar to our *Digital* implementation employing the same number of MAC units but based on a different dataflow, as a result of which it has lower throughput than our digital implementation. The area number of DianNao is after place-and-route, and thus includes metal wiring space as well, whereas those of our designs are based on synthesized logic gates only. But even after discounting the differences due to methodology, the compute density (i.e., synaptic connections per area) of DianNao is extremely low, making it unsuitable for neuromorphic architectures, where a large number of non-time-multiplexing neurons are expected. We note that the same weakness plagues our digital implementations as well. Eyeriss (Chen et al., 2017) is a well-known systolic array architecture to accelerate CNNs. While it is one of the most energy-efficient digital CNN accelerators, it has the lowest compute density (synaptic connections per area) in our comparison. That is because Eyeriss employs large PEs as well as many intra-PE registers and complex inter-PE connections, making it hard to scale to tens of thousands of PEs.

When the number of MACs is small, or when there is no area constraint, the DPS SC-CNN architecture achieves the best ADP, beating the neuromorphic architecture by about 2×. The neuromorphic architecture, on the other hand, shows very competitive ADP at a high throughput level. Note that the neuromorphic architecture has the exactly the same accuracy as the DPS SC-CNN, whose accuracy drop is <1% as shown in Table 1. All in all, these results indicate that our SC-based neural network is very flexible and scalable to accommodate various applications as well as area constraints.

### 5.7. Fault Tolerance

To evaluate the fault tolerance of our proposed schemes, we have performed an error injection experiment. For the fault model, we assume that random bit flip can occur at the input, as is done in a previous study on SC (Qian et al., 2011). The SRAM memories are assumed to be protected, such as using hardened logic or ECC (error correcting code). For a given fault rate *f*, we flip the bits of input registers, whose size varies depending on the scheme, with the same probability *f*. This fault model is integrated into the Caffe framework. No retraining is performed, but only inference, in the presence of faults.

**Figure 11:**
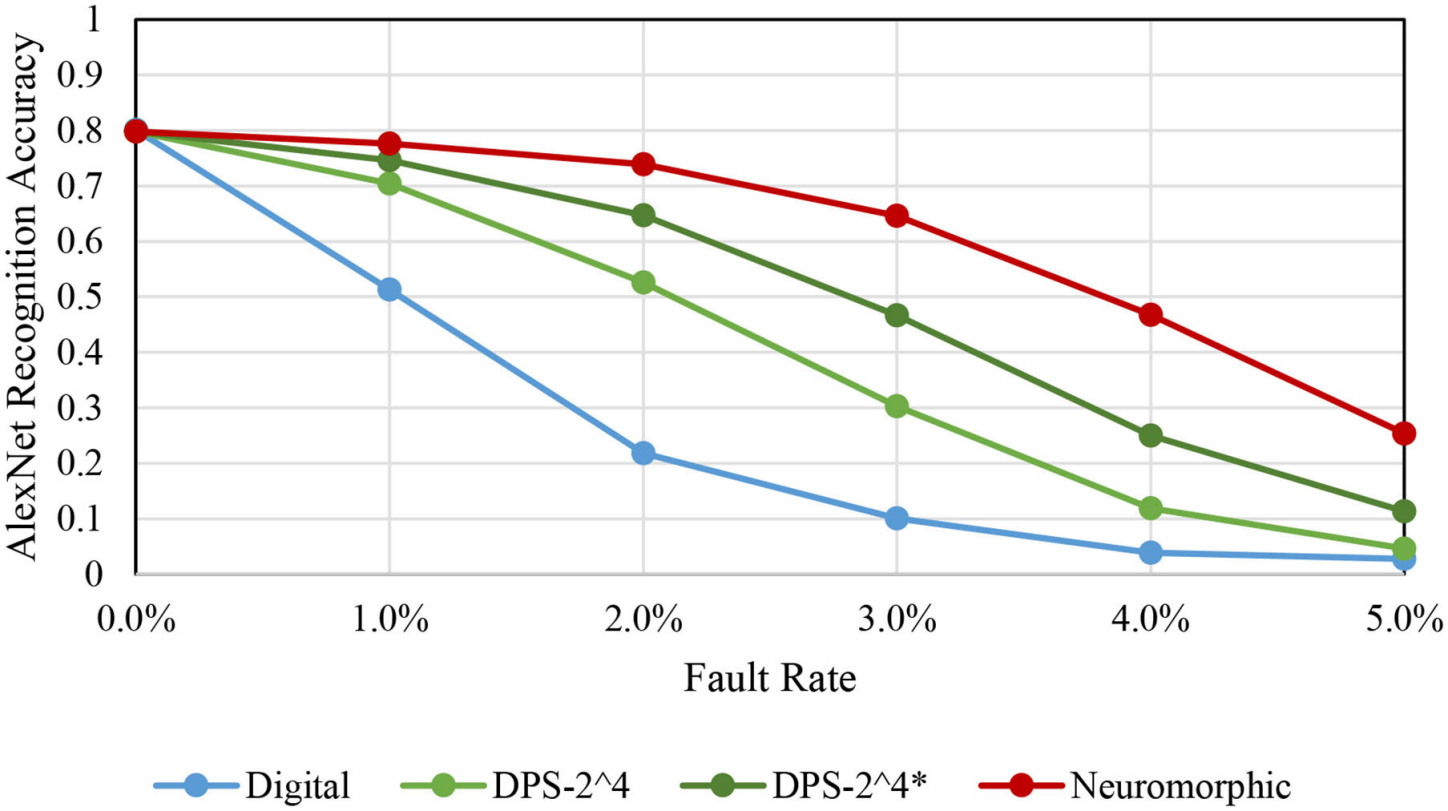
Fault tolerance comparison among different schemes, for AlexNet.

Figure 11 shows accuracy degradation for AlexNet as we vary fault rate. First, we observe that SC-based implementations show significantly higher fault tolerance than the conventional digital implementation, which agrees with previous studies (Qian et al., 2011; Zhakatayev et al., 2018). Second, there is quite a variance among the SC-based implementations. The neuromorphic implementation, which is based on bit-serial SC, shows the highest fault tolerance whereas the bit-parallel version, DPS-2^4, is less error resilient. There are a number of differences between the two architectures. One relevant fact is that the bit-parallel version performs a weighted bitcount operation to process multiple bits in parallel, which is more like digital logic than SC, and thus may contribute to its lower fault tolerance.

Another difference is that the neuromorphic architecture reloads input every cycle due to the tight SRAM integration. (The neuromorphic architecture has input registers too like the other architectures.) To test if this contributes to the higher fault tolerance, we test a variant of the DPS-2^4 scheme, denoted by DPS-2^4*, which is to reload input every cycle even when it is not necessary to do so. Our experimental result in Figure 11 clearly shows that input reloading helps. This may come as a surprise, but while input reloading does not lower the average number of faults in the circuit, it does lower the chance of having *correlated faults*, faults that occur at the same bit position of the input and therefore are more detrimental to the correctness of computation. The neuromorphic architecture has the least correlated faults, which helps achieve the highest fault tolerance.

### 5.8. Feature Comparison With Previous Work

In addition to performance numbers, our solution proposed in this paper has many important features as summarized in Table 3. The key factors that make our work much more efficient and accurate than the previous work are the combination of variable latency, dynamic precision (i.e., per-layer precision), multi-bit acceleration (crucial for larger CNNs), and HRS (which is specific to SC). In addition, ours has high fault tolerance inherent with SC.

**Table 3:** Feature comparison (O: supported, X: not supported).

**Feature**	**Digital**			**SC**			
	**DianNao (Chen et al., 2014)**	**Judd et al., 2016**	**Eyeriss (Chen et al., 2017)**	**Kim et al., 2016**	**Sim et al., 2017**	**Sim and Lee, 2017**	**Ours**
Large (≥5 Conv. layers) CNNs	O	O	O	X	X	X	O
Tile based	O	O	O	X	O	O	O
Per-CNN precision	X	O	X	X	X	X	O
Per-layer precision	X	O	X	X	X	X	O
Per-bit precision	X	O	X	O	O	O	O
Multi-bit acceleration	X	X	X	X	X	O	O
Variable latency operation	X	X	X	O	X	O	O
Half-range specialization	X	X	X	X	X	X	O

## 6. Conclusion

In this paper, we presented a bitstream-based neural network, which is a highly optimized and deterministic version of SC neural network. Thanks to many optimizations including dynamic precision scaling and half-range specialization in addition to the fundamental redesign of the SC multiplication operation, our SC-CNN can achieve both very high accuracy and high efficiency up to ImageNet-targeting CNNs. The SC-CNN owes some of its accuracy advantage to deep learning training algorithms, such as backpropagation. However, it has a distinct set of advantages over deep learning models due to SC, such as precision flexibility and error resilience. These advantages can be very useful, for instance, when designing a single piece of hardware that needs to efficiently support various neural networks with different precision requirements and when computation may not be reliable due to advanced semiconductor process scaling. The flexibility of precision comes with the challenge of optimizing it. Currently, our optimization flow is greedy and slow due to the retraining of SC-CNN. Finding better methods to determine optimal precision settings more quickly remains for future work.

## Data Availability Statement

The datasets generated for this study are available on request to the corresponding author.

## Author Contributions

HS did the design, implementation, and experimentation in the paper, and also made the initial draft of the paper. JL made critical contributions to the conception of the work, and extensive revisions of the paper. All authors contributed to the article and approved the submitted version.

## Conflict of Interest

The authors declare that the research was conducted in the absence of any commercial or financial relationships that could be construed as a potential conflict of interest.

## Footnotes

^1^Despite the deterministic nature of our SC-MAC, we retain the term, SC-MAC, in this paper because its operation is based on bitstreams. It also helps maintain consistency with previous work.

^2^https://github.com/BVLC/caffe/wiki/Model-Zoo
